# Visualizing surface marker expression and intratumoral heterogeneity with SERRS-NPs imaging

**DOI:** 10.7150/ntno.67362

**Published:** 2022-01-24

**Authors:** Lara K. Rotter, Naxhije Berisha, Hsiao-Ting Hsu, Kathleen H. Burns, Chrysafis Andreou, Moritz F. Kircher

**Affiliations:** 1 Department of Cancer Immunology and Virology, Dana-Farber Cancer Institute and Harvard University Medical School, Boston, MA, USA; 2 Department of Radiology, Memorial Sloan Kettering Cancer Center, New York, NY, USA; 3 Department of Neurology, Memorial Sloan Kettering Cancer Center, New York, NY, USA; 4 Department of Diagnostic and Interventional Neuroradiology, School of Medicine, Technical University Munich, München, Germany; 5 PhD Program in Chemistry, The Graduate Center of the City University of New York, New York, NY, USA; 6 Department of Nanotechnology, Advanced Science Research Center (ASRC) at the Graduate Center of the City University of New York, New York, NY, USA; 7 Department of Pharmacology, Memorial Sloan Kettering Cancer Center, New York, NY, USA; 8 Department of Pathology, Dana-Farber Cancer Institute and Harvard University Medical School, Boston, MA, USA; 9 Department of Electrical and Computer Engineering University of Cyprus, Nicosia, Cyprus; 10 Emphasis Research Centre, University of Cyprus, Nicosia, Cyprus; 11 Molecular Pharmacology Program, Memorial Sloan Kettering Institute, New York, NY, USA; 12 Department of Radiology, Weill Cornell Medical College, New York, NY, USA; 13 Department of Imaging, Dana-Farber Cancer Institute, Boston, MA, USA

**Keywords:** SERRS, Raman imaging, Raman Nanoparticles, Raman Spectroscopy, Brain tumor, Glioblastoma multiforme, Breast Cancer, Breast Cancer Metastasis, Tumor Heterogeneity, Surface marker expression, EGFR, HER2.

## Abstract

Cell surface marker expression in tumors dictates the selection of therapeutics, therapy response, and survival. However, biopsies are invasive, sample only a small area of the tumor landscape and may miss significant areas of heterogeneous expression. Here, we investigated the potential of antibody-conjugated surface-enhanced resonance Raman scattering nanoparticles (SERRS-NPs) to depict and quantify high and low tumoral surface marker expression, focusing on the surface markers epidermal growth factor receptor (EGFR) and human epidermal growth factor receptor 2 (HER2) in an intracerebral and peripheral setting with an inter- and intratumoral comparison of Raman signal intensities.

**Methods**: ICR-Prkdc <scid> mice were injected with glioblastoma, epidermoid carcinoma, or breast tumor cell lines intracerebrally and peripherally. SERRS-NPs were functionalized with cetuximab or trastuzumab and administered via tail vein injection. Raman imaging was performed 18 hours post-injection in excised tumors and *in vivo* through the skull. Tumors were then fixed and processed for immunohistochemical evaluation.

**Results**: Confirmed by MRI and immunohistochemistry for EGFR and HER2, our results demonstrate that antibody-conjugated SERRS-NPs go beyond the delineation of a tumor and offer clear and distinct Raman spectra that reflect the distribution of the targeted surface marker. The intensity of the SERRS-NP signal accurately discriminated high- versus low-expressing surface markers between tumors, and between different areas within tumors.

**Conclusion**: Biopsies can be highly invasive procedures and provide a limited sample of molecular expression within a tumor. Our nanoparticle-based Raman imaging approach offers the potential to provide non-invasive and more comprehensive molecular imaging and an alternative to the current clinical gold standard of immunohistochemistry.

## Introduction

Brain tumors, primary as well as metastases (secondary brain tumors), are potentially highly lethal in the setting of an oncological disease, with a median overall survival of 12-15 months for Glioblastoma multiforme (GBM), the most lethal primary brain cancer 1, and a median overall survival of 3-47 months for secondary brain tumors 2 but on average less than six months 3. In women, the most common cause of brain metastasis is breast cancer, with human epidermal growth factor receptor 2 (HER2) overexpression being a major risk factor for a reduced brain metastasis-free survival 3, 4 and a significantly reduced overall survival 3, 5. The diagnosis is established by surgical biopsy and is evaluated with a combination of histopathologic and molecular approaches. While imaging approaches are critical for guiding the biopsies and tumor resections, molecular imaging of specific biomarkers can enhance or enable the evaluation of residual disease or relapse. It can additionally help to characterize disease progression as tumors undergo changes with time, especially under the selective pressure of ongoing therapy 6-8. Molecular imaging also can reveal tumor heterogeneity, a hallmark of GBM. Heterogeneity in morphology and expression of GBMs is evident not only among different patients (intertumoral) but also within tumors of an individual patient (intratumoral) and may be a major factor in each patient's therapeutic response - as such, identification of such variations is essential 9, 10.

A single tumor can harbor a variety of subclones 10, 11, which may influence therapy response rate, progression-free and overall survival, as well as recurrence rate 10, 12-14*.* Cell surface receptors, especially those that can serve as targets for therapeutic agents, can be used to identify clinically significant tumor variations; one such example is the epidermal growth factor receptor (EGFR), which controls cell growth. EGFR has gained much attention as a clinical marker in GBMs and other cancers due to its reported correlation with survival and response to treatment 15-19. Aberrant expression of EGFR is observed in up to 60% of GBMs and leads to unrestrained cell growth and replication, as well as an increase in the cancer's aggressive potential. Receptor aberrancy is driven by abnormal gene amplification, receptor mutation (in particular the extracellular vIII domain), or both 18.

HER2 is a highly relevant surface marker in certain types of breast cancer, as it promotes cell proliferation; it is overexpressed in 25-30% of breast cancers 20 and is associated with a higher risk for brain metastasis and poor prognosis. Clinical outcomes, however, can be improved through targeted therapy with Trastuzumab, Trastuzumab-Pertuzumab, and Ado-Trastuzumab-Emtansine (T-DM1) 20-23. Hence, evaluation of the HER2 expression status, assessed chiefly by immunohistochemistry (IHC), is fundamental for patients with breast cancer.

Intratumoral heterogeneity and the degree of a surface marker's expression are thought to underlie the extensive therapy resistance and recurrence rate of GBMs and breast cancer 11, 24. However, assessing intratumoral heterogeneity and its effect on the therapeutic response is clinically challenging because biopsies are complex and invasive procedures, especially in the setting of intracranial (i.c.) tumors, and sample only a subset of the tumor's overall composition. In addition, a significant proportion of histologically assessed GBMs samples are at risk of being under-graded 25 and the evaluation of the HER2 expression in breast cancers is often subject to equivocal results 26, which may complicate treatment decisions and negatively impact prognosis.

Considering the limitations of biopsies 25-29, it is imperative to develop molecular imaging methods that can serve as a minimally invasive alternative with the capacity to visualize the tumor's entire landscape over time. The relevance of these methods is further spotlighted as new molecular targets emerge, offering personalized treatment options. Consequently, it would be valuable to develop non-invasive imaging modalities that can take advantage of novel molecular targets to determine the initial tumor composition and progression of tumor growth following treatment (e.g., chemo- or radiotherapy) 6-8, 30. A minimally invasive molecular imaging method can mitigate many of the risks associated with biopsies while enabling extensive and frequent tumor assessment.

Optical spectroscopy using surface enhanced Raman scattering (SERS) is a powerful technique with many applications in chemical detection and medical imaging 31. The use of surface-enhanced resonance Raman nanoparticles (SERRS-NPs) for clinical diagnostics has gained much attention recently 32-35, especially given the newer methodologies that allow minimally invasive detection and imaging 36-38. Molecular targeting is enabled by conjugating antibodies to the NPs and offers the potential for sensitive tumor detection and tumor phenotyping. Raman imaging that uses SERRS-NPs targeted with such antibodies may guide therapeutic options for more personalized medicine and enable treatment regimens that can be adapted to changes in tumor growth and biomarker expression 39. SERRS-NPs offer quantifiable signals in the form of distinct and quantifiable spectra stemming from adsorbed dyes, yielding a major advantage over traditionally used methods like IHC, which are harder to quantify and remain qualitative. While immunofluorescence can increase the quantitative sensitivity of conventional IHC, it nonetheless has significant drawbacks due to tissue autofluorescence, which negatively impacts its performance 40. SERRS-NPs could overcome this hurdle by providing quantitative information without the interference of autofluorescence 41.

Here we explore the possibility that SERRS-NPs can accurately delineate specific surface markers in tumors, specifically in primary and secondary brain tumors. In addition to delineating the tumor margins, we explore whether SERRS-NPs can quantify the expression of clinically important biomarkers in tumors. We hypothesize that tumor heterogeneity can be visualized based on the quantitative signal of the NPs. To address this hypothesis, we first tested whether targeted SERRS-NPs could quantify the expression of the molecular markers EGFR and HER2 in high- and low-expressing tumors in mice. Different cell lines were used to test both molecular markers. Raman imaging with targeted SERRS-NPs enabled not only the delineation of the main tumor but also was able to accurately differentiate between high- and low-expressing tumors. Additionally, among the high-expressing EGFR tumors, it was possible to visualize intratumoral heterogeneity regarding clusters of higher and lower EGFR expression levels.

## Material and Methods

### Materials

Unless otherwise noted, all chemicals were obtained from Sigma Aldrich (St Louis, MO). The antibodies cetuximab (Erbitux, Eli Lilly) and trastuzumab (Herceptin, Genentech) were received from the Memorial Sloan Kettering Cancer Center Pharmacy.

### Synthesis of SERRS nanoparticles

SERRS-NPs were synthesized as reported 42. Briefly, 60-nm gold nanostars were generated by adding 10 ml of a 20 mM gold chloride stock solution to one liter of 60 mM ascorbic acid at 4°C. The gold nanoparticles (GNPs) were collected by centrifugation and dialyzed (MWCO 3.5 kDa) for three days. To form SERRS-NPs, 5.4 ml of a dialyzed GNP dispersion was added to 45 ml absolute ethanol containing 900 µl ammonium hydroxide, then rapidly added to an already prepared Raman reporter/tetraethyl orthosilicate (TEOS) solution (90 µl of 25 mM Raman reporter IR780 perchlorate (IR780) in N,N-dimethylformamide, 13.5 ml 100% ethanol, and 2.25 ml TEOS). After 35 minutes, ethanol was added to quench the silication reaction; the SERRS-NPs were washed four times in ethanol.

### EGFR and HER2 targeted SERRS nanoparticle functionalization

The first step in functionalization introduced sulfhydryl (thiol) groups on the NP surface. The SERRS-NPs were redispersed in a solution of 850 µl 100% ethanol, 100 µl (3-mercaptopropyl)trimethoxysilane (3-MPTMS) and 50 µl deionized water, and warmed in a 70°C water bath for about two hours. The sulfhydryl-modified SERRS-NPs were collected by centrifugation, washed with ethanol and water, and concentrated to 3.5 nM. The heterobifunctional linker poly(ethylene glycol) (N-hydroxysuccinimide 5-pentanoate) ether N′-(3-maleimidopropionyl)aminoethane (PEG) in fivefold molar excess was incubated for 40 minutes with the cetuximab (0.5 mg/ml) or trastuzumab (1 mg/ml) antibodies in 10 mM 2-(N-morpholino)ethanesulfonic acid (MES) buffer (pH 7.1); the PEG-antibody solution was then washed once with phosphate-buffered saline (PBS) and two times with MES. To conjugate the purified antibodies and SERRS-NPs, 350 µl of 3.5 nM thiolated SERRS-NPs were added to the PEG-antibody solution and incubated for 30 minutes. Cetuximab-conjugated SERRS-NPs (cetuximab-SERRS-NPs) and trastuzumab-conjugated SERRS-NPs (trastuzumab-SERRS-NPs) were washed with deionized water and redispersed in 0.22-μm filter-sterilized 10 mM MES buffer (pH 7.3), at a final concentration of 3.5 nM.

### Characterization of SERRS nanoparticles

To characterize the NPs by transmission electron microscopy (TEM), samples of the functionalized NPs were deposited on carbon film-coated copper grids (300 Mesh, Electron Microscopy Sciences) and air-dried. Images were acquired at magnifications ranging from 50,000× to 250,000×, using a JEOL 1200EX (JEOL USA, Inc.) TEΜ operating at 80 kV. Concentration and size distribution of the SERRS-NPs were assessed by NP tracking analysis (NTA; NanoSight NS500; Malvern Instruments Inc.; Westborough, MA). SERRS signal intensity was measured using a Renishaw inVia Raman microscopy system (Renishaw, Hoffman Estates, IL) equipped with a piezo-controlled stage for micron-resolved spatial mapping, a 300-mW 785-nm diode laser, and a 1-inch charge-coupled device (CCD) detector with a spectral resolution of 1.07 cm^-1^. The SERRS spectra were collected through a 5× objective (Leica) and 1 s acquisition time at 0.05% laser power.

### Animal models

All animal experiments were approved by the Institutional Animal Care and Use Committee at the Memorial Sloan Kettering Cancer Center (# 16-09-013). I.c. and peripheral (flank, subcutaneous) tumors were induced by implanting various cell lines into four- to five-week-old ICR-Prkdc <scid> mice to yield tumors with varying expression levels of EGFR and HER2. For EGFR expression two mouse models were used, one with U87/U87EGFR tumors (flanks, n=2), and one with A431/TS895 tumors (flanks, n=3; brain, n=5). For HER2, HCC1954/MDA-MB-468 were used to induce peripheral tumors (n=2) as well as brain tumors (n=3). To induce high-expressing and low-expressing tumors, individual cell lines were injected into different loci of the same animal (either the two flanks or the two brain hemispheres).

To generate flank tumors, 10^6^ cells of each cell line were implanted. For the i.c. tumors, cells were implanted stereotactically, as listed in Table 1. Mouse models were produced for pairs of high- and low-expressing tumors. One hemisphere was injected with a high- and the other hemisphere with a low-expressing cell line, as follows: the needle tip was inserted 2.0 mm lateral to bregma, 3.5 mm below the dural surface, and then pulled up another 0.5 mm (to create a pocket for cells to settle). The final depth of the needle tip was 3.0 mm. The incidence and size of brain tumors were determined by weekly MRI scans (Bruker 7T MRI, Billerica, MA), starting on average two weeks after cell implantation. Brain tumors (i.c.) grew within four to eight weeks and peripheral tumors (flank) within two to four weeks.

### MRI

MR images were acquired on a dedicated small animal MRI scanner, consisting of a 7 Tesla superconducting magnet (Bruker Biospin Corp., Billerica, MA) and a gradient (Resonance Research Inc., Billerica, MA) with a clear bore size of 115 mm and maximum gradient amplitude of 640 mT/m. A custom-made 36-mm quadrature birdcage radiofrequency (RF) coil (Starks Contrast MRI coils Research Inc, Erlangen, Germany) was used for RF excitation and detection. Mice were immobilized by 2% isoflurane gas (Life Science, LLC, N Augusta, SC) in oxygen. Animal respiration was monitored with a small animal physiological monitoring system (SA Instruments, Inc., Stony Brook, New York). Scout images were acquired along three orthogonal orientations for animal positioning. To image the mouse brain, a brain coronal T2-weighted Rapid Acquisition with Relaxation Enhancement (RARE) fast spin echo sequence was used with the following parameters: 256×160 matrix, field of view (FOV) 3×2 cm, repetition time/echo time (TR/TE) of 1500/50 ms, 1 mm slice thickness, and 12 acquisitions in average. The total imaging time was six minutes.

### Raman imaging and signal quantification

When peripheral tumors reached a size of approximately 0.5-1.0 cm, the mice were injected via the tail vein with 350 µl of 3.5 nM cetuximab-SERRS-NPs or trastuzumab-SERRS-NPs, to image EGFR or HER2, respectively. The SERRS-NPs were allowed to circulate for 18-24 hours before mice were euthanized. Peripheral tumors were scanned via Raman imaging as fresh *ex vivo* tissue samples before fixation; brain tumors applied to sectioning were exposed to 4% formaldehyde for 30-45 minutes, and then sliced into two to three sections (thickness 0.5-1.5 mm). The brief fixation was necessary to stabilize the brain tissue and avoid its degradation during imaging. All Raman scans were carried out using our Raman system (see Methods) and Raman images acquired with the same focal plane (same objective lens), at 10-100% laser power, and 0.6-1s acquisition time in Map Image acquisition mode. A typical Raman scan took between 90-180 minutes. Raman images were analyzed using in-house software developed in Matlab (2017b). Regions of interest were determined via MRI of the whole brain *in vivo*, and histology of the brain tissue slices *ex vivo*. To remove fluorescent background signals from the Raman spectra, baseline fluorescence was subtracted with the Whittaker filter (λ=200 cm^‑1^), using PLS Toolbox v.8.0 (Eigenvector Research, Inc., Wenatchee, WA, USA).

### Histology

All tumors were fixed and embedded in paraffin at MSKCC. Tumor-bearing brains, brain-slices, and peripheral tumors were fixed in 4% paraformaldehyde overnight at room temperature, paraffin-embedded on a Leica ASP6025 tissue processor (Leica Biosystems). Immunostaining was performed at MSKCC and Dana-Farber/Harvard Cancer Center (DFHCC). At MSKCC, Paraffin-embedded tissue sections were cut at 5 μm and heated at 58°C for one hour. Samples were loaded into Leica Bond RX and pretreated with EDTA-based epitope retrieval ER2 solution (Leica, AR9640) for 20 minutes at 100°C. The rabbit monoclonal antibodies against pEGFR (Cell Signaling Technologies, Cat#4267BF, 4.4 µg/ml) were applied for 60 minutes and detected with Polymer Refine Detection Kit (Leica, DS9800). Antibody Leica Bond Polymer anti-rabbit HRP was used, followed by incubation with Refine Detection Kit Mixed DAB Refine for ten minutes, and Refine Detection Kit Hematoxylin counterstaining for ten minutes. After the staining, the sample slides were washed in water, dehydrated using ethanol gradient (70%, 90%, 100%), washed three times in HistoClear II (National Diagnostics, HS-202), and mounted in Permount (Fisher Scientific, SP15). Furthermore, 5 µm paraffin sections were processed for immunofluorescence on Leica Bond RX (Leica Biosystems) with 4.4 µg/ml EGFR Rabbit mab (Cell Signalling #4267BF) for 1 hour, using 10 minutes of 1:200 Tyramide Alexa Fluor488 detection (Life Technologies) on Protocol F. Sections were pretreated with Leica Bond ER2 Buffer (Leica Biosystems) for 20 minutes at 100°C before each staining. Stained sections were dehydrated, mounted with Mowiol, and digitally scanned on a Pannoramic Confocal (3dHistech) microscope, using a 40× water objective. Slides were digitally scanned with Leica Aperio. At DFHCC, IHC was performed on the Leica Bond III automated staining platform using the Leica Biosystems Refine Detection Kit. Anti-EGFR (Cell Signaling Technology, # 4267, clone D38B1; 1:50 dilution) antibody was run with a 30-minute EDTA antigen retrieval. Antibody against HER2 (Neomarkers, # 9103-SO-A, clone SP3; 1:40 dilution) was run with a 30-minute citrate antigen retrieval.

## Results

### Synthesis of antibody-conjugated SERRS-NPs

**Figure 1:**
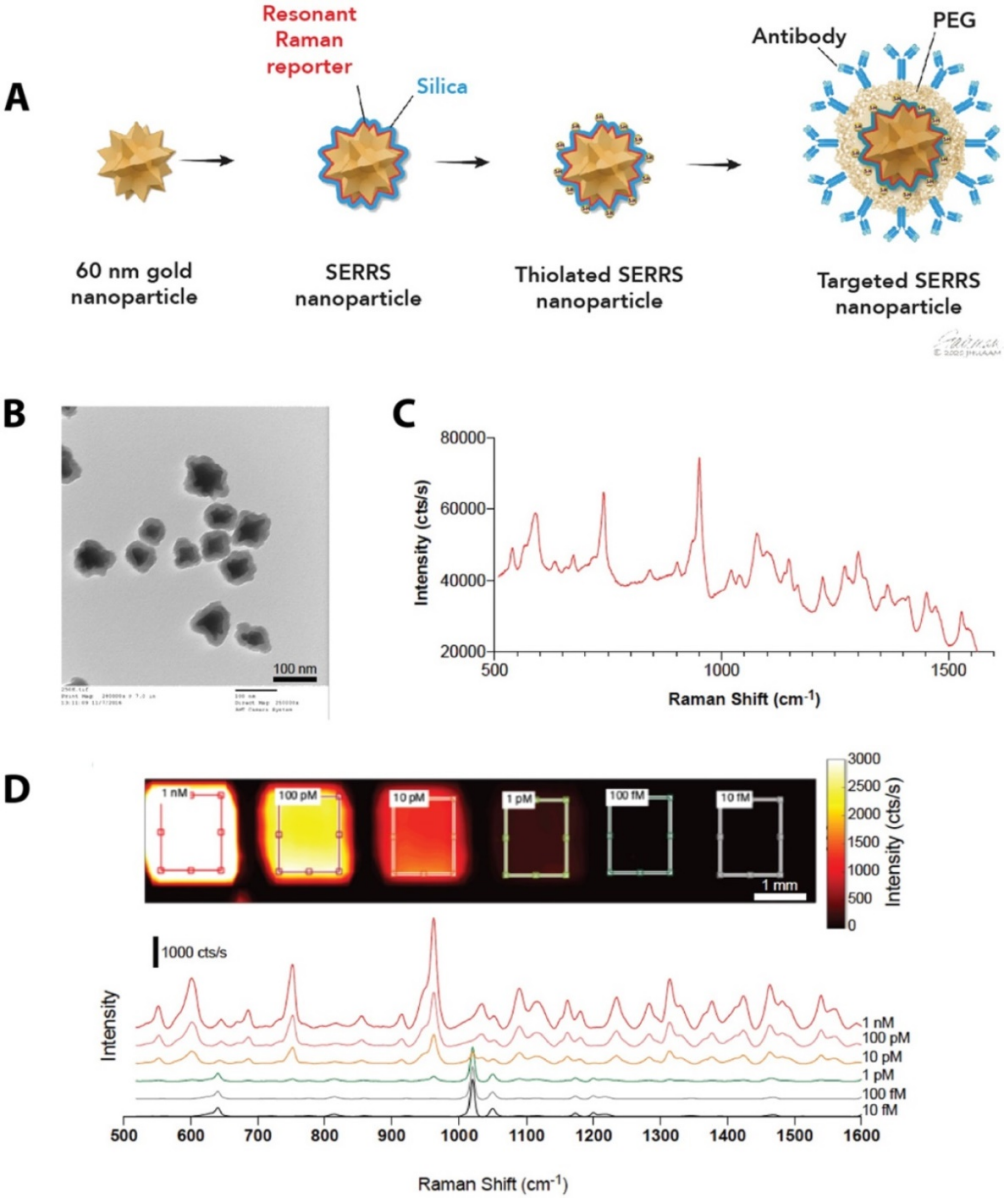
** Synthesis of SERRS-NPs.** (**a**) Schematic illustration of the targeted SERRS-NPs synthesis. Sixty nanometer gold nanostars were synthesized. A resonant Raman reporter molecule was added during silication. The silicated nanostars underwent further modification with thiolation and were functionalized via a heterobifunctional PEG crosslinker with either cetuximab or trastuzumab. (**b**) Quality control was performed with transmission electron microscopy (TEM) imaging, to assess the silica shell formation. Scale bar = 100 nm. (**c**) SERRS spectra of the cetuximab-SERRS-NPs with several distinct spectral peaks. (**d**) Dilution series showed a limit of detection of 1 pM after baseline subtraction. The spectra are shifted for clarity.

We synthesized SERRS-NPs as described 42 (also see Methods), with gold nanostar cores, the infrared dye IR780 perchlorate as a Raman reporter, and a silica shell, and functionalized each via a heterobifunctional PEG crosslinker (schematized in Figure 1A) with either cetuximab or trastuzumab. Quality control was performed with TEM imaging (Figure 1B) to assess the core morphology and the silica shell formation. Throughout the experiments, the concentration of the SERRS-NPs was evaluated by NP tracking analysis. The unique fingerprint of the synthesized SERRS-NPs shows several distinct spectral peaks (Figure 1C). The raw spectrum includes a substantial fluorescence background in addition to the sharp Raman bands; imaging of a dilution series showed a limit of detection of 1 pM after removal of this background. The intensity of the characteristic peak at 950 cm^-1^ can be used to detect the presence and quantity of nanoparticles as it demonstrates a non-linear, monotonically increasing relation with the nanoparticle concentration. The peak detected at 1020 cm^-1^ along with the other various minor peaks are derived from the plastic material of the 384-well plate and become apparent when the signal of the SERRS-NPs is low (Figure 1D).

### Antibody-conjugated SERRS-NPs enable quantitative assessment of the surface markers EGFR and HER2, and delineate high versus low EGFR/HER2- expressing tumors

**Figure 2:**
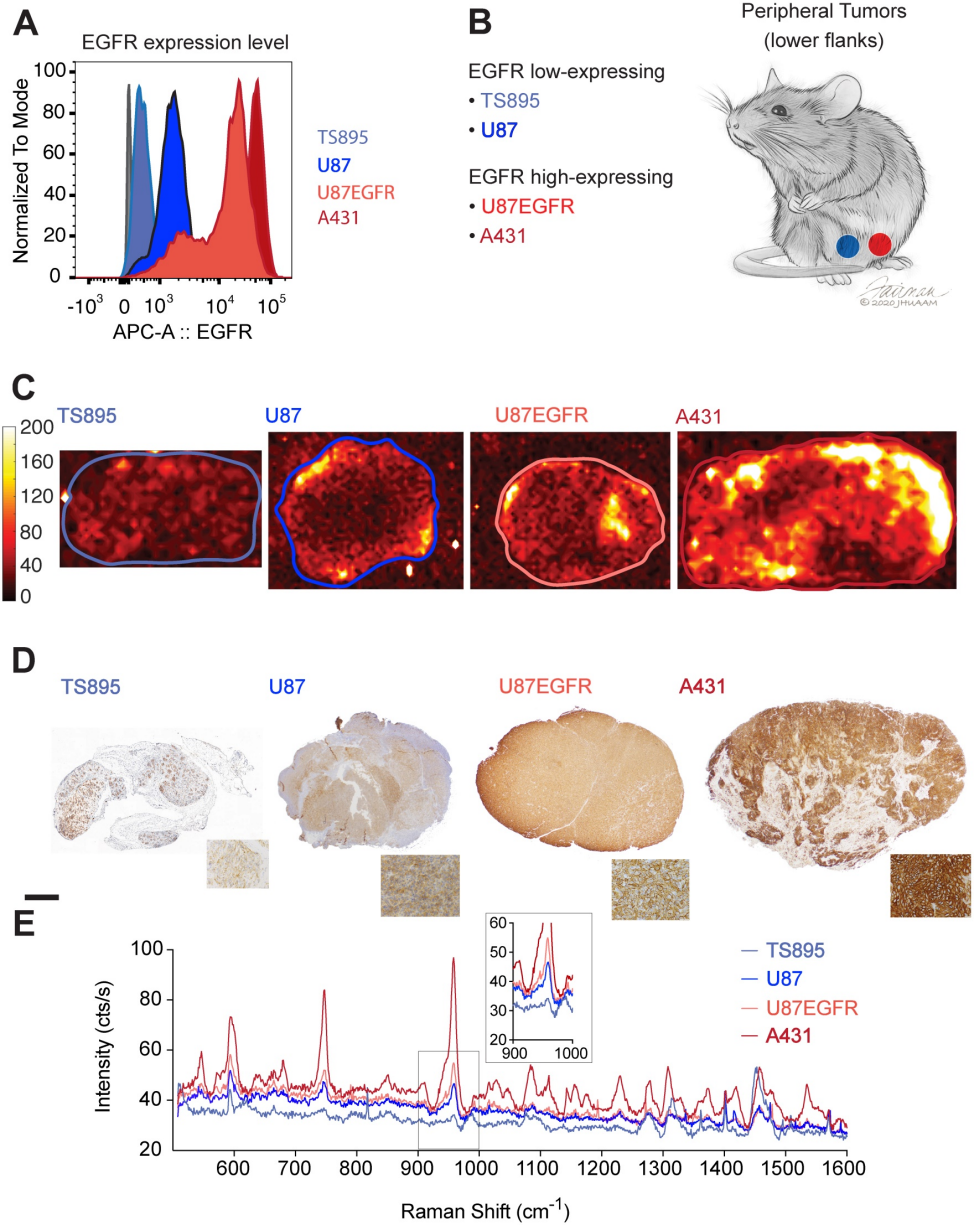
** EGFR expression in peripheral tumors.** (a) Four cell lines were selected with varying expression of EGFR determined by flow cytometry. (b) ICR scid mice received flank injections. One flank was injected with a low-expressing, and the other flank with a high-expressing EGFR cell line. (c) Raman maps of the 950 cm^-1^ peak with EGFR-targeted SERRS-NPs in excised tumor sections (thickness 2-4 mm) reveals the distribution of the biomarker. Low-expressing tumors n=8, high-expressing tumors n=10. (d) IHC staining against EGFR shows the increasing expression levels tumor sections, corroborating the flow cytometry data. The scale bar corresponds to 1 mm. (e) Averaged Raman spectra from the tumors corresponding to the areas enclosed by the lines shown in (c). The intensity of the Raman signal corroborates the EGFR expression level determined by flow cytometry and IHC. The inset shows the SERRS-NP characteristic peak from the lower-expressing tumors.

ICR scid mice were injected with GBM and epidermoid carcinoma cell lines that displayed high (A431 and U87EGFR cells) or low (TS895 and U87) EGFR expression. Flow cytometry confirmed decreasing EGFR expression from A431 to U87EGFR to U87, with TS895 showing the lowest EGFR expression (Figure 2A). Mice were injected with high EGFR-expressing cells in one flank and low EGFR-expressing cells into the opposite flank (Figure 2B) to induce peripheral tumors. Cetuximab-SERRS-NPs were then injected into the mice, allowed to circulate for 18-24 hours, after which the mice were euthanized, tumors were harvested, halved, and immediately subjected to Raman imaging, which showed that tumors with high EGFR expression exhibit higher intensity than their EGFR low-expressing counterparts (Figure 2C). In particular, the difference in Raman intensity between the cell lines A431 and TS895 was more evident than that between the cell lines U87 and U87EGFR, corroborating the expression levels for each, derived from the initial flow cytometry analysis. After imaging, tumors were paraffin-embedded and sequential sections were cut and processed with hematoxylin and eosin (H&E) and IHC staining for EGFR. The IHC staining also confirmed high EGFR expression for the tumors derived from the A431 and U87EGFR cells and low expression for tumors derived from TS895 and U87 cells (Figure 2D). The differences in Raman signal between high- and low-expressing tumors were found to corroborate with the flow cytometry measurements and to follow the same trend as established via IHC, considering the non-linear intensity curve of SERRS-NPs as shown in Figure 1D. The Raman spectra within each tumor (as indicated by the boundaries in Figure 2C) were averaged to produce representative spectra for each tumor. These spectra revealed that the levels of EGFR expression are also represented in the intensity of the averaged spectral signal. Raman spectra of the high-expressing EGFR tumors (derived from A431 cells; dark red) versus the low-expressing EGFR tumors (derived from TS895 cells; light blue) were more substantially different than were spectra of the high-expressing EGFR tumors (derived from U87EGFR cells; bright red) versus that from the low-expressing EGFR tumors (derived from U87 cells; dark blue) as shown in Figure 2E, verifying our initial flow cytometry analysis.

**Figure 3:**
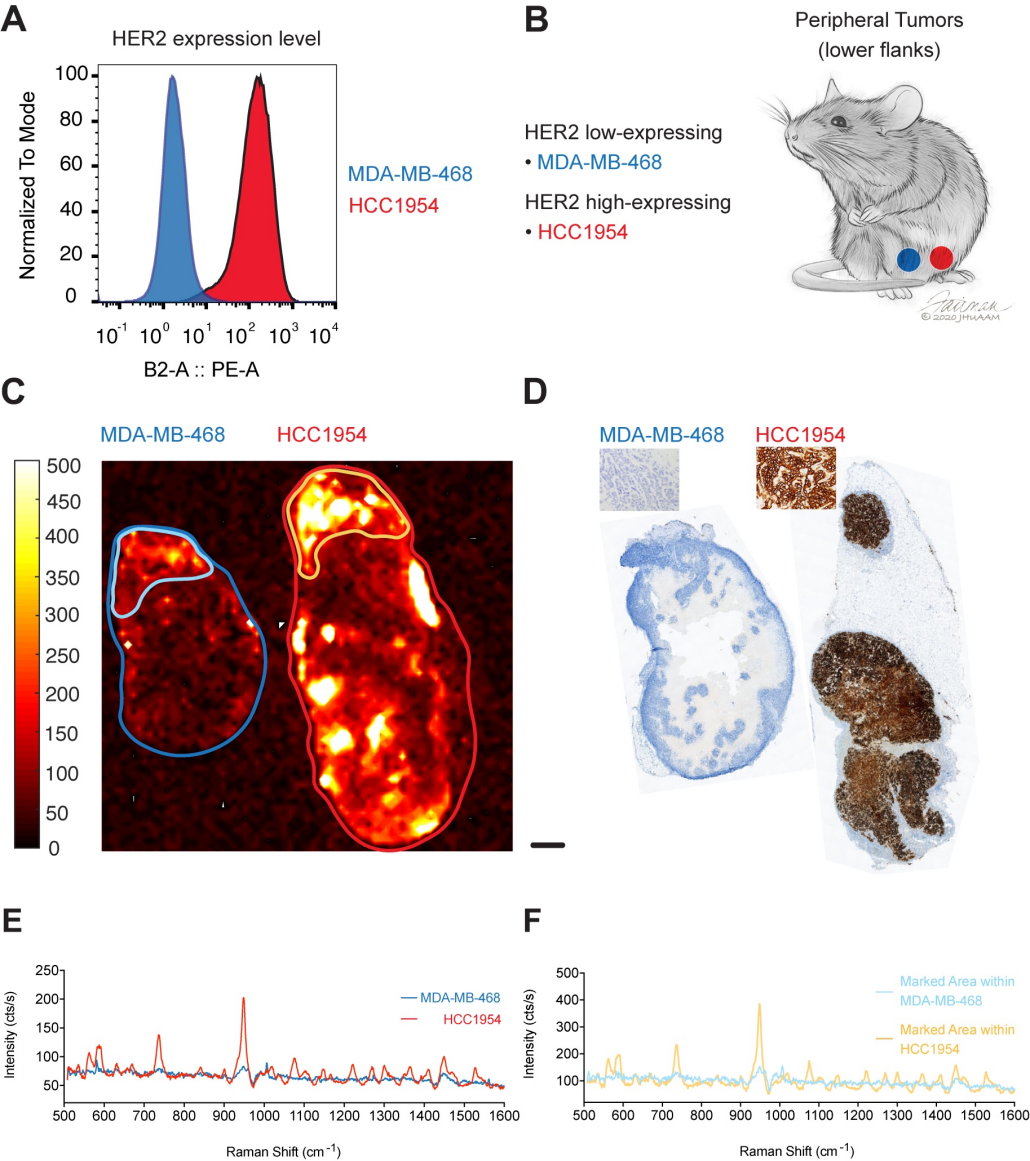
** HER2 expression in peripheral tumors.** (a) Two cell lines were used with high and low expression of HER2, determined by flow cytometry. (b) Cell lines were used to induce tumors bilaterally in ICR scid mice. (c) Raman imaging with trastuzumab-SERRS-NPs shows the distribution of the NPs in freshly excised tumors. Low-expressing tumors n=2, high-expressing tumors n=2. (d) IHC for HER2 reveals a differential expression in the tumors. Scale bar = 1 mm. (e) Raman spectra averaged within the regions shown in (c) correspond to the high and low expression of the tumors, as determined by flow cytometry and IHC. (f) Raman spectra of selected areas of relatively high intensities in the low-expressing tumor (Figure 1C, light blue) and in the high-expressing tumor (Figure 1C, yellow).

To test if our targeted SERRS-NPs would display the same potential in tumors with another target, we used our methodology to image HER2. HCC1954 cells were used as the HER2 high-expressing breast tumor cell line, and MDA-MB-468 cells as the HER2 low-expressing cell line. Expression levels of the biomarker were confirmed via flow cytometry (Figure 3A). As above, ICR scid mice were injected in their flanks with breast tumor cells that expressed high or low levels of HER2 (Figure 3B) and allowed to grow for two to four weeks. Trastuzumab-SERRS-NPs were injected intravenously (i.v.) via the tail vein and after 18-24 hours, the animals were euthanized, tumors harvested and bisected, and immediately subjected to Raman imaging. As above, the imaging showed that tumors with high HER2 expression showed higher intensity levels than their low expression counterparts (Figure 3C). The tumors were then paraffin-embedded, sequential sections cut, processed with H&E, and immunostained for HER2. The IHC also confirmed high HER2 expression in the tumors derived from the cell line HCC1954, and low HER2 expression for tumors from the MDA-MB-468 cell line (Figure 3D). Analysis of the respective Raman spectra (averaged over the areas indicated in Figure 3C) showed that different levels of HER2 expression were also detected in the Raman spectra intensity levels (Figure 3E). We observed moderate Raman signals from areas of the low-expressing tumor that appear negative on the IHC slide. This signal may correspond to low levels of non-specific uptake of the NPs, or from HER2-positive areas at a different depth at that locus. Depending on the optical configuration, Raman imaging acquires data from a focal plane with a thickness of up to several millimeters while histology slices are limited to an average slice thickness of 5-7 µm, and therefore, depending on the sampled area, may miss relevant regions of surface marker expression. Further analysis of the moderate-intensity area within the overall HER2 low-expressing tumor showed a significantly lower Raman signal intensity (outlined in light blue in Figure 3C, Raman spectrum in light blue in Figure 3F) when compared to a high-intensity area within the high-expressing HER2 tumor (outlined in yellow in Figure 3C, Raman spectrum in yellow in Figure 3F).

**Figure 4:**
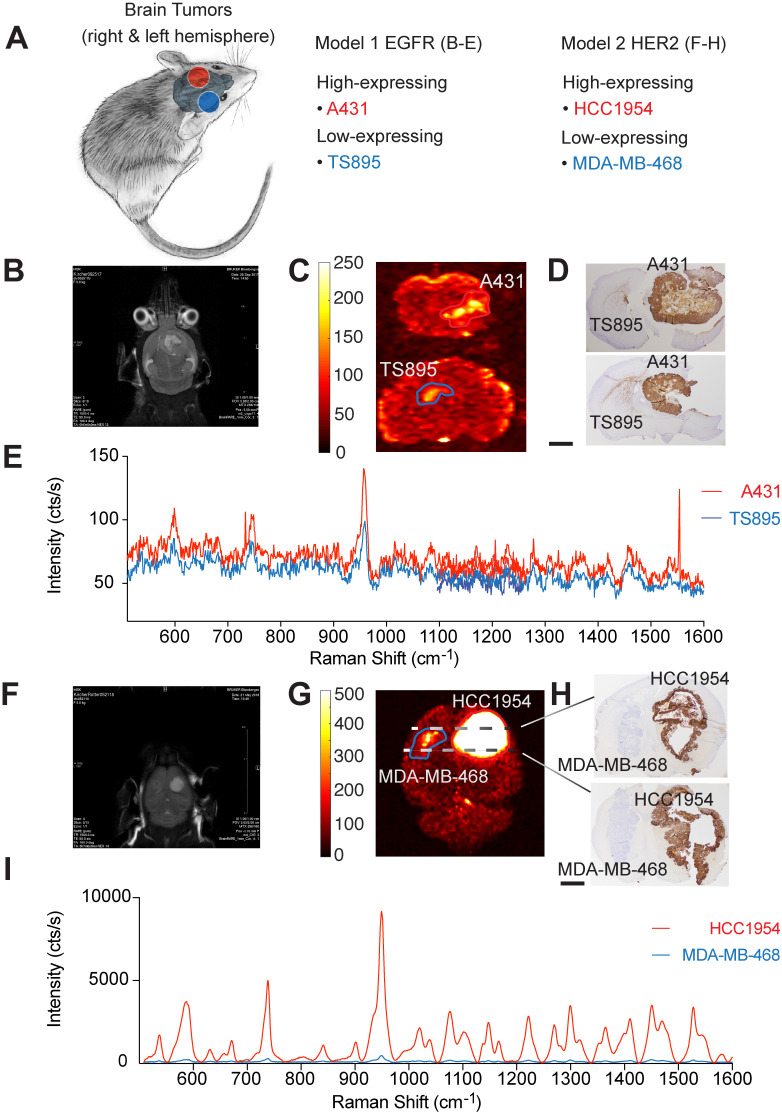
** Imaging biomarker expression in brain tumors.** (a) Orthotopic primary and secondary tumor models were induced by stereotactically implanting, in both brain hemispheres, cells that express EGFR or HER2 at varying levels. (b) MRI image, five weeks after injecting A431 (n=5) and TS895 (n=4) cells. (c) Raman imaging of brain sections from mice injected with cetuximab-SERRS-NPs reveals the tumors, based on EGFR expression. (d) IHC staining reveals the differential expression of EGFR in the two tumors. Scale bar = 1 mm. (e) Average Raman spectra from the areas indicated in (c) reveal the low and high expression of EGFR in the tumor. (f) MRI image at four weeks post-injection with HCC1954 (n=3) and MDA-MB-468 cells (n=3). (g) Raman imaging of whole excised brain using trastuzumab-SERRS-NPs documents the location of the two tumors. (h) IHC staining for HER2 in sections, corresponding to the dotted lines in (g), show the differential expression of the biomarker. Scale bar = 1 mm. (i) The averaged Raman signal intensity from the areas indicated in (g) shows the difference in expression level of HER2 between the two tumors.

These data advocate for the potential of targeted SERRS-NPs to reveal different surface expression patterns accurately as well as quantitatively in various types of tumors. To evaluate if this potential of our targeted SERRS-NPs also applies to i.c. located tumors, we injected ICR scid mice in one hemisphere with high-expressing and in the other hemisphere with low-expressing tumors for two biomarkers (Table 1, and Figure 4A). Results of the EGFR-targeted imaging are shown in Figure 4B-E, and for HER2 in Figure 4F-I.

For EGFR-expressing tumors, MRI was performed at one- to two-week intervals, starting at four weeks post-implantation (Figure 4B). Brain tumor-bearing mice were injected with cetuximab-SERRS-NPs and euthanized 18-24 hours later. *Ex vivo* Raman imaging was performed on whole brains or their coronal sections. The imaging showed higher intensity in tumors with high EGFR expression than in their EGFR low-expressing counterparts in the contralateral hemisphere (Figure 4C). The imaged brains were then fixed, paraffin-embedded, sectioned sequentially, stained with H&E, and processed for IHC staining to visualize EGFR. IHC staining confirmed a high expression pattern of the brain tumor derived from A431 cells, which express high levels of EGFR, and a low expression pattern of the brain tumor derived from the TS895 cells that express low levels of EGFR (Figure 4D). Analysis of the respective Raman spectra (averaged over areas of highest intensities shown in Figure 4C) shows that different levels of EGFR expression are also represented at various intensities of the Raman spectra: higher intensity for the EGFR high-expressing brain tumor (red spectrum) and lower intensity for the EGFR low-expressing brain tumor (blue spectrum) (Figure 4E).

These results show that targeted SERRS-NPs can differentiate patterns of surface expression of EGFR in the periphery as well as in the brain and led us to test the same breast tumor cell lines but in an i.c. breast cancer metastasis model. ICR scid mice were injected in one hemisphere with the high-expressing HER2 cell line HCC1954, and in the other hemisphere with the low-expressing HER2 cell line MDA-MB-468 (Figure 4A). MRI imaging was performed at one- to two-week intervals, starting at four weeks post-injection (Figure 4F). Animals were injected with trastuzumab-SERRS-NPs and then euthanized 18-24 hours later. *Ex vivo* Raman imaging of the brain (fixed in formaldehyde for approximately 45 minutes and cut into 0.5-1.5 mm-thick slices) showed that tumors with high HER2 expression had markedly higher intensity than their low-expressing HER2 counterparts in the contralateral hemisphere (Figure 4G). The brain slices were paraffin-embedded, sequential sections cut, stained with H&E, and immunostained for HER2. The IHC staining confirmed the high expression pattern of the simulated breast cancer metastases derived from the HER2 high-expressing cell line HCC1954, and the low expression pattern of the ones derived from the HER2 low-expressing cell line MDA-MB-468 (Figure 4H). The corresponding Raman spectra (averaged over the areas indicated in Figure 4G) documented that those different levels of HER2 expression are also represented, at dramatically differing intensity levels of the Raman spectra, with a higher intensity for the HER2 high-expressing simulated breast cancer metastasis (red spectrum) and a lower intensity for the HER2 low-expressing counterpart (blue spectrum) (Figure 4I). This dramatic difference in Raman intensity correctly recapitulates the findings of IHC, which show that the HCC1954 tumor has consistently and markedly high HER2 expression. The difference in intensity levels between the Raman spectra for the HER2 model was much more pronounced than the difference for the EGFR model. This observation corresponds to the respective IHC staining showing a positive IHC staining for the EGFR high-expressing tumor, while very strong staining was observed for the HER2 high-expressing tumor.

**Figure 5:**
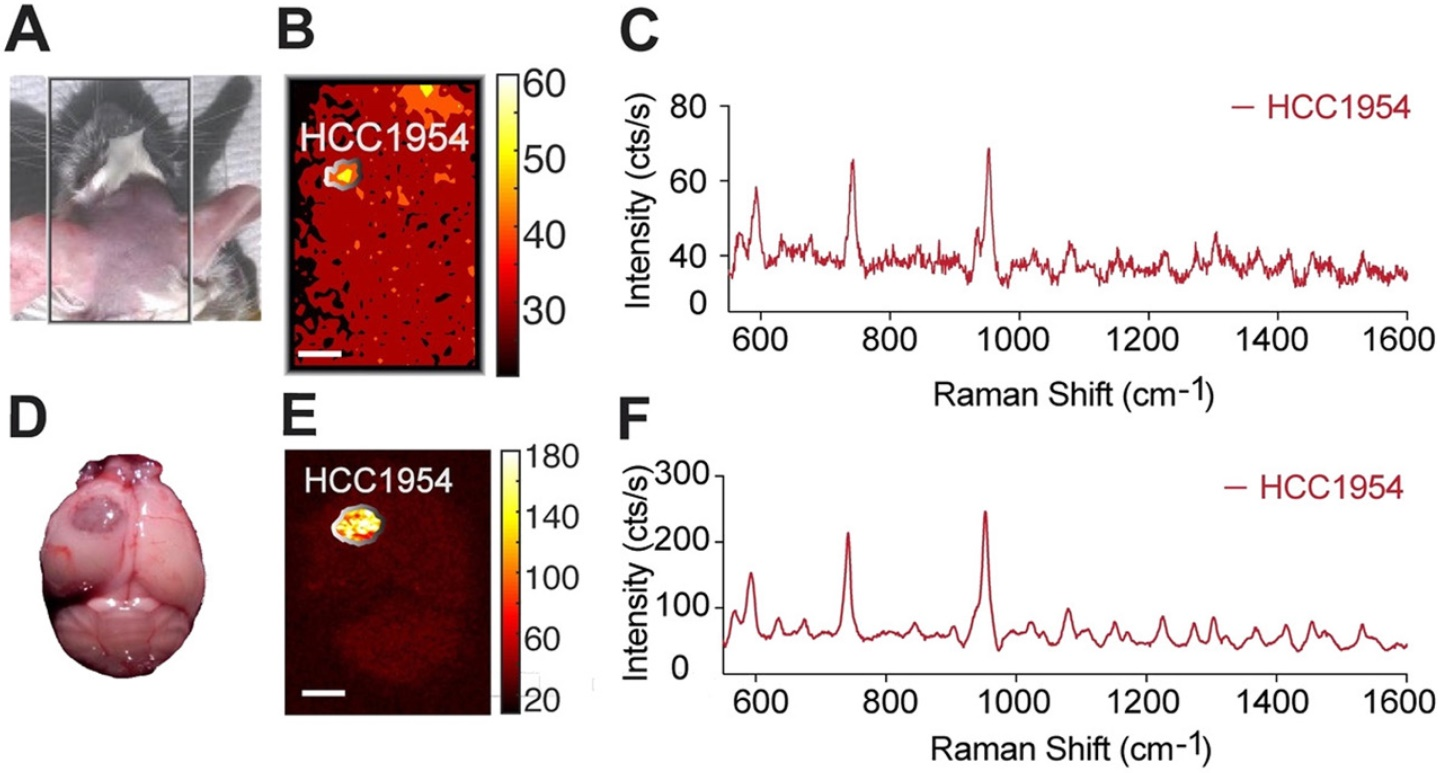
***In vivo* non-invasive imaging through the skull.** (a) After trastuzumab-SERRS-NPs injection, the head skin was shaved, and the mouse was subjected to Raman imaging. (b) Raman map of the 950 cm^-1^ peak indicates the presence of the SERRS-NPs through the skin and skull. (c) The Raman spectrum from the area indicated in (d) matches the signature of the trastuzumab-SERRS-NPs. (d-f) The results of (b-c) were confirmed with *ex vivo* Raman imaging, after removing the brain. Scale bars = 1 mm.

To assess whether our methodology can be used for minimally invasive imaging, we also tested its potential to detect an i.c. tumor *in vivo,* through the intact skin and skull of a mouse model of a HER2-positive tumor. As above, the tumor was induced by implanting HCC1954 cells in one hemisphere of the mouse, and HER2-targeted SERRS-NPs were synthesized and administered. Before imaging, the mouse was anesthetized with an intraperitoneal injection of ketamine (15 mg/ml) and xylazine (1.5 mg/ml) cocktail (10 ml/g). As shown in Figure 5, the tumor could be delineated *in vivo* through the intact skin and skull.

Collectively, these results point to the potential of targeted SERRS-NPs to accurately image different surface expression patterns in various types of tumors, in the periphery as well as within the brain.

### EGFR-targeted cetuximab-SERRS-NPs enable quantitative assessment of the EGFR surface marker and of intratumoral heterogeneity

**Figure 6:**
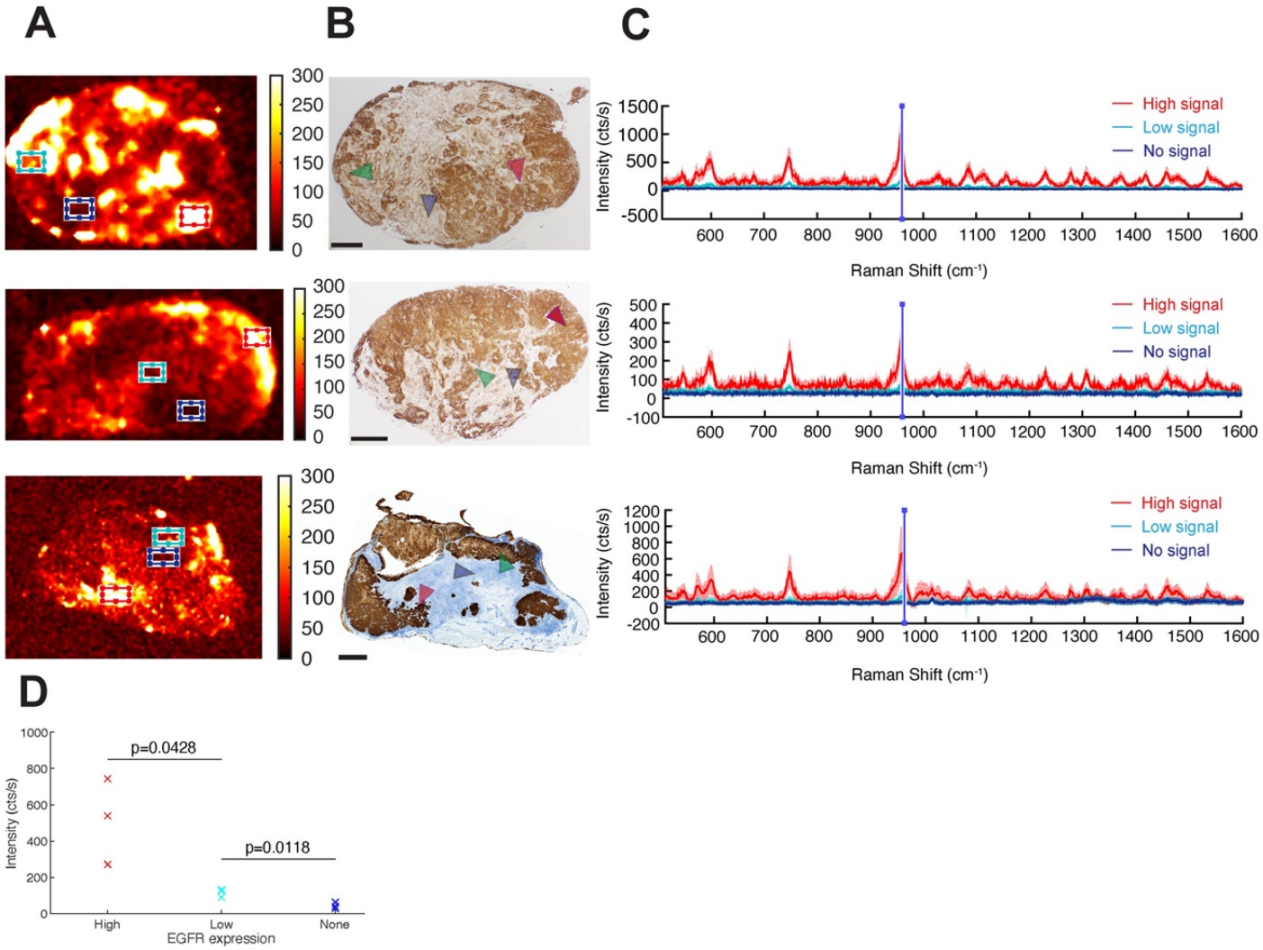
** Intratumoral heterogeneity in EGFR high-expressing tumors.** (a) Different areas within the same tumor exhibit different levels of cetuximab-SERRS-NP signal. Three independent tumors are shown as examples. (b) IHC reveals the inhomogeneous expression of EGFR within the tumors (n=8). The expression pattern is comparable to the corresponding Raman maps. (c) Raman spectra provide a quantitative indication of the expression level of EGFR in the different areas indicated by boxes in (a) and by areas in (b). (d) The two-variable Student t-test shows differences in mean intensities are statistically significant with p-values of 0.0428 between high- and low-expressing areas, and 0.0118 between low-expressing and negative areas. The scale bars correspond to 1 mm.

Having demonstrated that targeted SERRS-NPs can distinguish high versus low surface marker expression in tumors, we next explored if the NPs could be used to evaluate the intratumoral heterogeneity of surface marker expression by analyzing the Raman images of EGFR high-expressing tumors (Figure 6A) and confirming the findings by paraffin-embedding the Raman-imaged tumors, sectioning and immunostaining them for EGFR (Figure 6B). Raman signals from different tumor areas were averaged (Figure 6C). High, low, and not detectable SERRS-NP signals were collected from red, cyan, and blue areas, respectively (Figure 6A), and the marker expression level was confirmed via IHC (see arrowheads). The average spectra in each case were calculated after baseline subtraction from 70 individual point spectra within the rectangular regions shown, with dimensions of 1500 × 900 µm^2^. For each spectrum, the mean intensity of the band between 950 and 960 cm^-1^ (including the main peak of the Raman spectrum) represented the indicative intensity. Areas of high EGFR expression had intensities >200 cts/s, whereas regions of low expression had intensities around 100 cts/s (Figure 6D). Areas considered to have no EGFR expression showed intensities of around 50 cts/s, corresponding to background signal and noise rectification produced by baseline subtraction. The reported counts depend on the applied imaging system configuration, including optics and acquisition time, and therefore are arbitrary; but when used consistently, they provide quantitative information to the corresponding qualitative data collected from IHC. A two-variable Student t-test indicated that the differences in mean intensities are statistically significant, with p-values of 0.0428 between high- and low-expressing areas, and 0.0118 between low-expressing and negative areas. The analysis shows that Raman signals from targeted SERRS nanoprobes can be used to differentiate areas of high, low, and no biomarker expression (Figure 6D).

## Discussion

Our findings are a promising step toward surmounting some of the hurdles related to inter- and intra-tumoral heterogeneity and obstacles pertaining to biopsies and subsequent IHC staining. As discussed above, tumoral heterogeneity is of great importance for a variety of tumors and may influence crucial aspects in a patient's disease process, like therapy response and recurrence rate, as well as progression-free and overall survival 10, 12-14. Tumoral heterogeneity of surface markers is mostly assessed by IHC. IHC requires either an invasive biopsy or surgery to obtain tissue samples, which, however, represent only a restricted area and are potentially not representative of a tumor's entire composition 25-27. Furthermore, IHC depends on the expertise of the performing laboratory and the evaluating pathologist 28, 29. Lastly, longitudinal monitoring of a tumor's development in response to the selection pressure during therapy, which may lead to the acquisition of new mutations and alter the tumor's surface marker profile 6, 7, would require serial biopsies, exposing the patient repetitively to biopsy-related risks like bleeding and infection while still being restricted to the sampled area of a tumor. Markers like EGFR and HER2 are clinically important targets in cancer treatment but might either be missed (if their surface expression is underestimated based on a limited biopsy) or lose their potential as a successful treatment target (if their expression changes with treatment). Here we show that targeted SERRS-NPs have the potential to overcome these limitations, and also offer various advantages. For example, their “Raman-Fingerprint”, which does not originate from endogenous biomolecules, offers high sensitivity and specificity 43-45. As contrast agents, SERRS-NPs are significantly more photostable than are the fluorochromes that are currently in use 40, and the sulfhydryl-modification of the NPs allows them to be conjugated with most antibodies (or another targeting element) via a simple thiol-maleimide bond 42. Most relevantly, targeted SERRS-NPs can be injected i.v. and used with minimally invasive imaging technology to visualize the entire tumor, along with the differential distribution of targeted surface markers within the tumor. Moreover, SERRS-NPs consist of gold and silica, which are inert and do not lead to significant toxicity, as has been shown in extensive toxicity studies 46: thus, the imaging may be repeated multiple times longitudinally, and can enable the composition and expression levels of a whole tumor to be monitored, from initial diagnosis through treatment as well as during post-treatment surveillance. As a result, we can identify changes in the tumor's surface expression pattern that might render an initial treatment targeted against a suitable surface marker no longer appropriate and adapt the treatment according to how the tumor develops. Raman imaging also showed the potential for differentiating EGFR high- and low-expressing tumors using EGFR-targeting SERRS-NPs. Our findings highlight the potential of cetuximab-SERRS-NPs in differentiating not only high- versus low-expressing EGFR tumors, but also in recapitulating the degree of EGFR expression.

Previous work has shown that the so-called enhanced permeability and retention (EPR) effect 47 facilitates a baseline uptake of NPs and is observed in a variety of solid tumors, like breast, prostate, and pancreatic cancer as well as in sarcomas and pre-cancerous lesions 43. Although the degree of the EPR effect may differ between cancer types, as well as between mice and human patients, studies have shown that molecularly targeted NPs outperform untargeted NPs applications 48-50 and can increase their avidity leading to an enhanced residence time and an increase in their association constant by four to five orders in magnitude 51. Our data show that the SERRS-NPs can not only delineate different surface expression patterns in different types of tumors in the periphery and the brain but can also be used to address the heterogeneity of surface expression of EGFR within a tumor, emphasizing the potential of these NPs for future clinical applications in tumor diagnostics and therapy monitoring.

Using the approach we present here, Raman spectra reveal the biomarker distribution in the entire tumor, spanning several millimeters in depth, with no sample preparation. Acquiring samples for flow cytometry and IHC via biopsy requires a relevant degree of invasiveness beforehand and nevertheless only samples a small area of the tumor. The current technique does not allow for imaging through the human skull, but we were able to show that an accurate brain tumor delineation is possible in an in vivo setting in mice. With the advances already taking place in hand-held Raman scanners 38, 52, 53, endoscopic Raman scanner developments 54, 55, and importantly, methods using spatially offset optics 56, it may become possible to observe advances in the imaging of i.c. tumors.

In summary, the NP-based Raman imaging technology described here offers a minimally invasive process that holds great promise for visualizing expression patterns of molecules on a tumor's entire surface, represents a new method for improving quantitation of biomarker expression *in vivo*, and thus is an essential step in overcoming the hurdles of ordinary biopsies and bringing us closer to a more personalized patient treatment.

## Conclusion

Understanding a tumor's composition and its adaptation under treatment is critical, particularly when considering the numbers of cancer patients whose tumors become refractory to current state-of-the-art treatments, even when using personalized therapies. Characterizing the architecture of a tumor requires complex phenotyping before the treatment is initiated, and also at regular intervals during the treatment. To facilitate such monitoring while also reducing the need for repetitive biopsies, we have shown here that high- and low-expressing EGFR and HER2 tumors can be differentiated with the use of cetuximab- or trastuzumab-conjugated SERRS-NPs, respectively. To our knowledge, this is the first report of a targeted uptake of SERRS-NPs, which in turn leads to a “yes” or “no” answer, but also of the capability of targeted SERRS-NPs to accurately represent and quantify the level of expression of surface markers in different tumor regions. Progress in multiplexed imaging studies will yield targeted SERRS-NPs with the potential to retrieve more quantitative information about surface markers. Our technique is amenable to *in vivo* imaging through the intact skin and skull of a mouse, signifying the potential for non-invasive imaging. Possible clinical applications that enable Raman Spectroscopy through the intact human skin and skull, however, will require additional enhancements of the intensity of SERRS-NPs, as well as an improvement of the imaging speed and penetration depth. Further studies will also be needed to fully exploit the potential of SERRS-NPs in defining surface markers. Our findings yield the first steps toward realizing a minimally invasive assessment of a tumor's composition, and the information that can be captured with longitudinal observations on how it is altered over time.

## Figures and Tables

**Table 1:** Cell lines used for generating intracranial tumor mouse models.

Biomarker expression level	Cell line	Type of cell line	# of cells implanted/ hemisphere
EGFR low-expressing	TS895	GBM	500 000
	U87	GBM	500 000
EGFR high- expressing	A431	Epidermoid carcinoma	500 000
	U87EGFR	GBM	20 000
HER2 low- expressing	MDA-MB-468	Breast Cancer	20 000
HER2 high- expressing	HCC1954	Breast Cancer	500 000
